# Sensitivity and specificity of Cobas TaqMan MTB real-time polymerase chain reaction for culture-proven Mycobacterium tuberculosis: meta-analysis of 26999 specimens from 17 Studies

**DOI:** 10.1038/srep18113

**Published:** 2015-12-09

**Authors:** Nobuyuki Horita, Masaki Yamamoto, Takashi Sato, Toshinori Tsukahara, Hideyuki Nagakura, Ken Tashiro, Yuji Shibata, Hiroki Watanabe, Kenjiro Nagai, Kentaro Nakashima, Ryota Ushio, Misako Ikeda, Kentaro Sakamaki, Takashi Yoshiyama, Takeshi Kaneko

**Affiliations:** 1 Department of Pulmonology, Yokohama City University Graduate School of Medicine. 3-9, Fukuura, Kanazawa, Yokohama, Japan; 2 Department of Biostatistics, Yokohama City University Graduate School of Medicine. 3-9, Fukuura, Kanazawa, Yokohama, Japan; 3 Division of Respiratory Medicine, Japan-Anti-Tuberculosis Association Fukujuji Hospital. 3-1-24, Matsuyama, Kiyose, Tokyo, Japan

## Abstract

Since 2010, studies on the diagnostic accuracy of COBAS TaqMan MTB (CTM) have been frequently reported with an unignorable discrepancy. The key inclusion criterion for this systematic review was original studies that could provide sufficient data for calculating the sensitivity and the specificity of CTM for *M tuberculosis* (TB) or *M tuberculosis* complex. The reference test was *Mycobacterium* culture. We used bivariate model for meta-analyses. Of the 201 candidate articles, we finally identified 17 eligible articles.Concerning the respiratory specimens, 1900 culture positive specimens and 20983 culture negative specimens from 15 studies were assessed. This provided the summary estimate sensitivity of 0.808 (95% CI 0.758–0.850) and the summary estimate specificity of 0.990 (95% CI 0.981–0.994). The area under curve was 0.956. The diagnostic odds ratio was 459 (95% CI 261–805, I^2^ 26%). For the smear positive respiratory specimens, the sensitivity was 0.952 (95% CI 0.926–0.969) and the specificity was 0.916 (95% CI 0.797–0.968). For the smear negative respiratory specimens, the sensitivity and the specificity were 0.600 (95% CI 0.459–0.726) and 0.989 (95% CI 0.981–0.993), respectively. The diagnostic accuracy was poorer for the non-respiratory specimens, than for the respiratory specimens, but was acceptable. We believe that the information obtained from this study will aid physicians’ decision making.

In 2013, approximately 1.5 million people died of *Mycobacterium tuberculosis* (*M. tuberculosis*). To provide appropriate antibiotic treatment, rapid and accurate detection of the *M. tuberculosis*, which has been a clinical challenge for more than a century, is crucial^1^. Classical acid-fast stain and mycobacterium culture still play a central role in the diagnosis of tuberculosis (TB), although they have considerable limitations: the acid-fast stain lacks specificity and the culture needs weeks of the incubation time^2^,^3^. Nucleic acid amplification tests, such as polymerase chain reaction (PCR) which was developed in 1983, are now an indispensable tool in the TB diagnosis as it provides quick and specific results to clinicians within hours^2^,^3^.

The COBAS Amplicor PCR system was the first commercially available automated nucleic acid amplification analyzer^4^. For the clinical evaluation, the COBAS Amplicor presented excellent sensitivity for smear-positive specimens, moderate sensitivity for smear-negative specimens, generally good specificity, and acceptable PCR inhibition rate^5^,^6^,^7^. Later, Roche Diagnostics also developed a novel real-time PCR system, COBAS TaqMan, which is more widely accepted now. For the diagnosis of TB, COBAS TaqMan MTB (CTM) focuses on a segment of the 16S rRNA gene^8^. Since 2010, studies on the diagnostic accuracy of CTM have been frequently reported with an unignorable discrepancy^9^,^10^,^11^,^12^,^13^,^14^,^15^. Some previous studies suggested that these heterogeneous results may be caused by inconsistent specimen types and smear status^9^,^10^,^11^. Even though manufacturer instruction limited CTM application only for the respiratory specimens^16^, researchers often evaluated the diagnostic accuracy of CTM for the non-respiratory specimens. It is because we have limited resources for extra-pulmonary TB. Therefore, in the current systematic review and meta-analysis, we assessed the diagnostic accuracy of CTM for the non-respiratory specimens in addition to the respiratory specimens.

## Methods

We conducted this meta-analysis following pre-specified protocol (UMIN000018272) following the Preferred Reporting Items for Systematic Reviews and Meta-Analyses (PRISMA) statement^17^ and standard guidelines for systematic review of diagnostic test accuracy^18^. Quality of included studies was assessed by Quality Assessment of Diagnostic Accuracy Studies-2 (QUADAS2)^19^. Approval of Institutional Review Board was waived for reviewing nature of the current study.

## Study Search

Two investigators (HN, NK) independently and systematically searched PubMed, PMC, Cochrane Library, and Web of Science database. Our last search for these medical data base was done on June 13^th^, 2015. Hand search was conducted by checking references of already included studies and published guidelines. In addition, Google Scholar was searched. Articles in authors’ reference list were also considered candidates. The inclusion criterion for this study was original studies that could provide sufficient data for calculating the sensitivity and/or the specificity of CTM for *M. tuberculosis* or *M. tuberculosis* complex. The reference test was culture proven *M. tuberculosis*. The composite reference standard combining the results of several reference tests was not allowed. The sensitivity was defined as true positives/(true positives + false negatives). The specificity was defined as true negatives/(true negatives + false positives). Cobas TaqMan probes other than CTM was excluded. For instance, TaqMan probe designed for IS6110 was excluded. No age limit was set. No publication-date limit was set. No language restriction was set, as long as an article provided sufficient data in English-written title, abstract, figure, or table. Besides full articles, short articles were also considered for inclusion. Report with sample size less than 20 were excluded. HIV status was not considered. We did not included a study that reported the respiratory and the non-respiratory specimens collectively as we have to evaluate them separately. A study that reported only the sensitivity or the specificity was excluded because we could not perform the bivariate meta-analysis for such a study. Duplicate use of the same data was examined carefully and excluded.

## Search formulas

We used following formulas. PubMed: (TaqMan OR (Cobas real PCR)) AND tuberculosis AND (sensitivity OR specificity OR positive OR negative OR (predictive value)). Cochrane library: (tuberculosis OR mycobacterium OR TB) AND (diagnosis OR sensitivity OR specificity OR PCR OR TaqMan). Web of Science: TaqMan AND tuberculosis AND (sensitivity OR specificity OR (predictive value)). PMC: TaqMan MTB.

## Cobas TaqMan MTB preparation

Regardless of specimen types, i.e. the respiratory or the non-respiratory specimens, specimens were generally liquefied and decontaminated with N-Acetyl-L-Cysteine-Sodium Hydroxide (NALC/NaOH), according to manufacturer instruction^16^. However, some investigators used other preparation methods (Table 1).

## Outcome

The numbers of positive and negative results were counted specimen based, and not person based.

CTM rarely output invalid results suggesting PCR inhibition, contamination, internal control signal being out of range, or low positive control. Therefore, samples with invalid results were excluded preceding the sensitivity and specificity evaluations because the manufacturer instruction recommended to repeat the entire run for the PCR procedure, and because most original researches reported only the numbers of positive and negative results. The pooled invalid rate was estimated separately from diagnostic accuracy.

We divided specimens in each study based on specimen types because the respiratory and the non-respiratory specimens should be evaluated independently. This was because CTM was originally designed only for the respiratory specimens^19^. We classified the specimens into the respiratory and the non-respiratory categories based on the description in each original report. The respiratory specimens included sputum, bronchial/tracheal aspirate, bronchial/tracheal lavage, broncho-alveolar lavage. The non-respiratory specimens included lymph node, articular fluid, ascites fluid, abscess/pus, urine, cerebrospinal fluid, other tissue, and other body fluids. Gastric fluid and pleural fluid were classified either into the respiratory or the non-respiratory specimen in each original report.

In addition to classification based on the specimen origin mentioned above, we performed subgroup analyses focusing on the smear status because we had acknowledged that the smear status affects the diagnostic accuracy of CTM.

## Statistics

We drew a paired forest plot and bivariate model summary receiver operating characteristics curves (SROC), which showed potential trade-offs between the sensitivity and the specificity. The summary estimate of the sensitivity and the specificity was also obtained from the bivariate model^20^.

To assess the overall accuracy, we calculated diagnostic odds ratio (DOR) using DerSimonian-Laird random-model and the area under SROC curve (AUC) using the bivariate model. We estimated the pooled invalid rate using the simple random model with generic inverse valiance.

The heterogeneities for the DOR among studies and between subgroups were evaluated using I^2^ statistics with the following interpretation: I^2^ = 0, no heterogeneity; 0 < I^2^ < 25, mild heterogeneity; 25 ≤ I^2^ < 50, moderate heterogeneity; 50 ≤ I^2^ < 75, strong heterogeneity; 75 ≤ I^2^ < 90, considerable heterogeneity; 90 ≤ I^2^, extreme heterogeneity^21^. Publication bias was not evaluated as this is not usually recommended in the meta-analysis for the diagnostic test accuracy^18^.

The paired forest plot and the random-effect model meta-analysis were performed using Reviewing Manager ver. 5.3 (Cochrane Collaboration, Oxford, UK). Following commands of “mada” package on free software R were used: “madauni” for DOR and “reitsma” for SROC, AUS and summary estimate for the sensitivity and the specificity^22^,^23^.

## Results

### Study search

**Figure 1:**
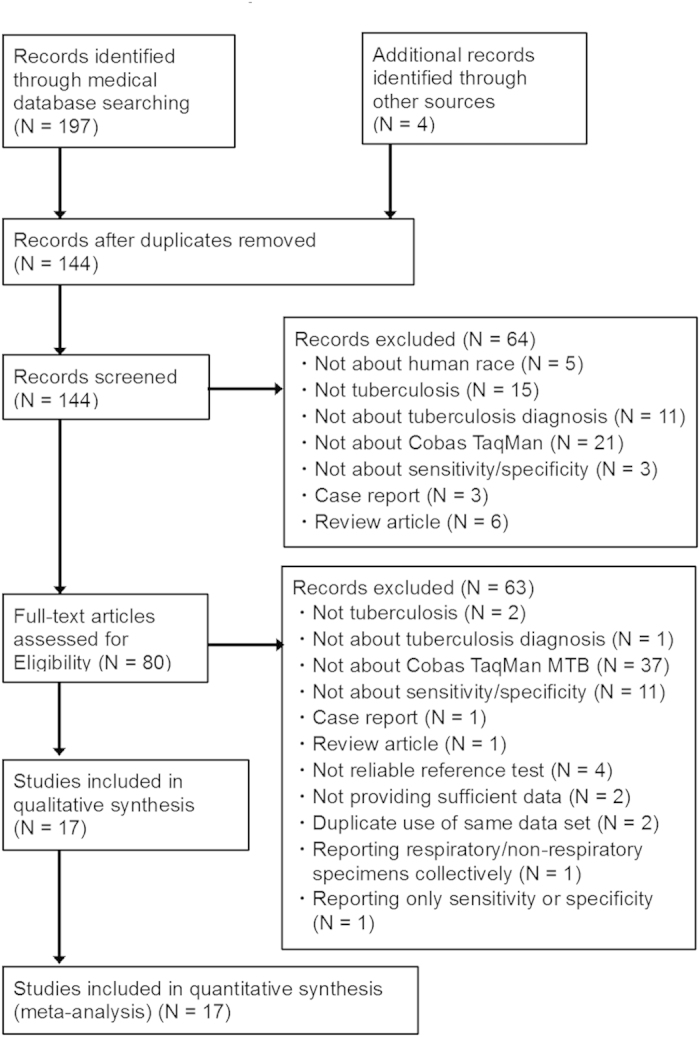
The Preferred Reporting Items for Systematic Reviews and Meta-Analyses (PRISMA).

Of the 201 candidate articles, we finally identified 17 eligible articles (Fig. 1). Notably, two candidate articles were excluded due to overlapping data^24^,^25^. The finally eligible 17 articles included 16 English-language articles and one Korean-language article (Table 1). There were 16 full-length articles, and one non-full-length article. Most of the studies were reported from high-income or upper-middle income Asian countries, namely, Korea. From EU region, there was one article each from Germany, Italy, Spain, Sweden, and Switzerland. One article focused only on adult cases, three articles declared inclusion of both adult and child cases, and the others did not mention the age limitation. Three articles used frozen specimens and seven clearly wrote that the studies were conducted prospectively (Table 1).

The number of specimens in an article ranged from 72 to 6772 with a median of 619 specimens. The total number of specimens that provided valid results amounted to 26999, which consisted of 22883 respiratory specimens and 4116 non-respiratory specimens (Table 1, Supplementary Table 1). In addition, our data included 68 specimens that provided invalid results.

### Quality evaluation

We assessed the quality of the studies using the QUADAS2. In two studies, samples were used from already diagnosed TB and non-TB individuals. These studies were considered to have a case-control design; thus, these two studies have a high risk of bias concerning patient selection. One study did not provide sufficient description regarding the methodology concerning the reference tests, mycobacterial culture, and subsequent TB confirmation. This study with insufficient description was considered to have a high risk of bias concerning reference test. No other domain had a high risk of bias or a high applicability concern (Supplementary Figure 1).

### Respiratory specimen

**Figure 2:**
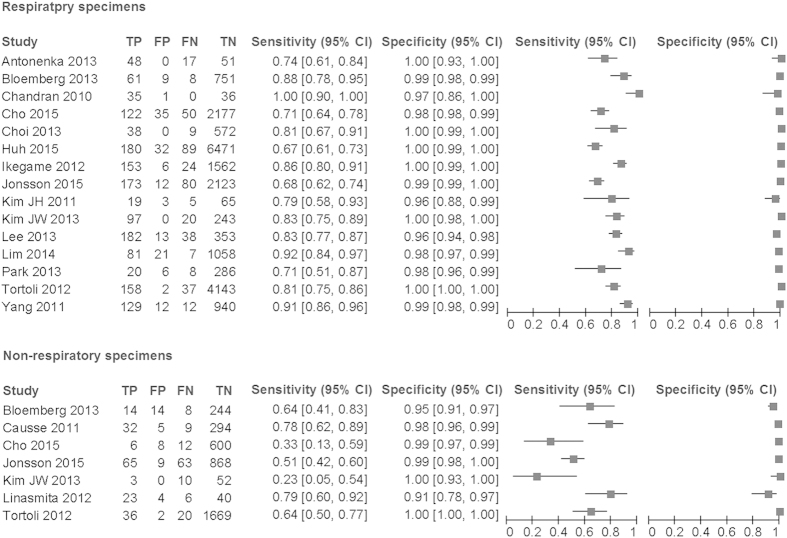
The paired forest plot for the diagnostic accuracy. TP: true positive. FP: false positive. FN: false negative. TN: true negative.

**Figure 3:**
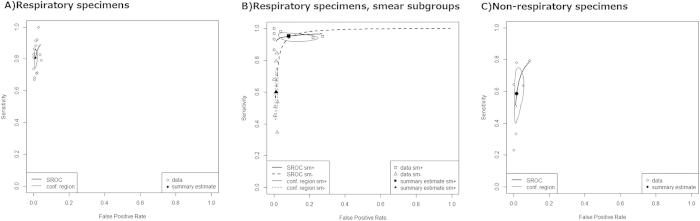
The summary receiver operating characteristics curves. SROC: summary receiver operation characteristics curve. conf. region: confidence region. sm+, smear positive. sm−, smear negative.

From 15 studies, 1900 culture positive specimens and 20983 culture negative specimens were assessed (Table 2, Figs 2 and 3A). This provided the summary estimate sensitivity of 0.808 (95% CI 0.758–0.850) and the summary estimate specificity of 0.990 (95% CI 0.981–0.994). The AUC was 0.956. The DOR was 459 (95% CI 261–805) with a moderate heterogeneity of 26%.

We performed subgroup analyses based on the smear status (Figs 2 and 3B). When focused on the smear positive specimens, seven studies with 566 culture positive specimens and 255 culture negative specimens were assessed. This yielded AUC of 0.961 and DOR was 269 (95% CI 66–1104) without heterogeneity. Concerning the smear negative specimens, the same seven studies provided data for 498 culture positive specimens and 11123 culture negative specimens. These yielded the AUC of 0.971 and the DOR of 132 (95% CI 71–243) with least heterogeneity of 14%. These data suggested that CTM provided the similar overall diagnostic accuracies for both the smear positive and the negative specimens. However, the summary estimate sensitivity and specificity differed largely based on the smear status. For the smear positive specimens, the sensitivity was 0.952 (95% CI 0.926–0.969) and the specificity was 0.916 (95% CI 0.797–0.968). For the smear negative specimens, the sensitivity and the specificity were 0.600 (95% CI 0.459–0.726) and 0.989 (95% CI 0.981–0.993), respectively.

As a sensitivity analysis, we conducted a subgroup analysis based on whether the *M. tuberculosis* was confirmed after culture or not. For 13 studies with the TB confirmation, the sensitivity, the specificity, the AUC and the DOR were 0.790, 0.990, 0.954, and 401, respectively. For two studies without the TB confirmation, the sensitivity, the specificity, the AUC and the DOR were 0.895, 0.991, 0.966 and 978, respectively. The differences between subgroups were not so large. Overestimation of the specificity due to the lack of *M. tuberculosis* confirmation was not strongly suggested.

### Non-respiratory specimens

According to seven studies with 307 culture positive non-respiratory specimens and 3809 culture negative specimens, the diagnostic accuracy was poorer than that for respiratory specimen but still acceptable. The AUC was 0.898 and the DOR was 86 (95% CI 34–217, I^2^ 7%). The summary estimate sensitivity and specificity were 0.586 (95% CI 0.437–0.721) and 0.984 (95% CI 0.955–0.994), respectively (Table 2, Fig. 3C).

A subgroup analysis for the non-respiratory specimens based on the smear status was not conducted because only one study reported the diagnostic accuracy based on the smear-status subgroup.

### Invalid rate

**Figure 4:**
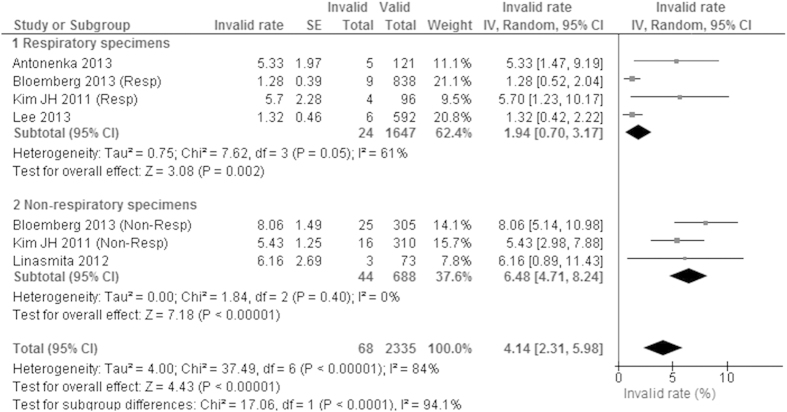
The forest plot for invalid rate. Valid: sum of positive and negative results.

Number of specimens with the invalid data was described in five original studies with 2403 specimens. The pooled invalid rate was 4.1% (95% CI 2.3–6.0%) with considerable heterogeneity of 84% (Fig. 4). In a subgroup analysis, the invalid rate was higher for the non-respiratory specimens (6.5%) than for the respiratory specimens (1.9%) (P < 0.001).

## Discussion

To our knowledge, this is the first systematic review and meta-analysis for CTM diagnostic accuracy. According to the AUC and the DOR, diagnostic accuracy of CTM was excellent for the respiratory specimens (Table 2). The invalid rate was also as low as 1.9% for the respiratory specimens (Fig. 4). Even though both the diagnostic accuracy and the invalid rate were poorer for the non-respiratory specimens, these results were acceptable for practice (Table 2, Fig. 4). Worldwide, nearly 10 million people, including a million children, fell ill with TB every year. We strongly hope CTM, which can provide the quick accurate result, will contribute to early and accurate diagnosis of TB.

In the current study, we conducted a subgroup analysis comparing the smear positive and the negative respiratory specimens. Heterogeneity assessed by I^2^ statics between these subgroups was generally not strong (Table 2), which meant that CTM has the similar diagnostic accuracy for both subgroups. However, the summary estimates of the sensitivity and the specificity of CTM for the culture-proven TB clearly depended on the smear status. It is not surprising given the difference of bacterial load depending on the smear status. Positive sputum smear may reflect the abundant bacterial load probably above a detection threshold. The CTM summary estimate sensitivity for the smear-positive respiratory specimens was 0.952, which suggested that negative result in this category is reliable. Thus, a CTM negative for a smear-positive respiratory specimen strongly suggests the presence of non-TB mycobacterium or a false-positive smear that is sometimes obtained on Auramine-Rhodamine staining. The moderate CTM summary estimate sensitivity for the smear-negative specimen of 0.600 meant that CTM potentially have inability to detect TB in 40% of the smear-negative culture-positive respiratory specimens. The CTM summary estimate of specificity for smear-negative respiratory specimens of 0.989 meant that a CTM positive in this category result strongly suggests TB diagnosis. The summary estimate specificity for the smear-positive respiratory specimens of 0.916 was significantly lower than that for the smear-negative respiratory specimens, which meant that, based on definition of specificity, there are non-negligible number of CTM false positives for smear-positive specimens. However, this situation is caused by combinations of “smear positive, CTM positive, and culture negative,” or “smear positive, CTM positive, culture positive for non-TB Mycobacterium”. In such situations, we should consider the possibility of culture false negative, misclassification of TB into non-TB Mycobacterium, and dead TB bacilli. Cross reactivity for non-TB mycobacterium may be another concern. However, CTM cross reactivity for non-TB mycobacterium is believed very rare^26^.

We compared the accuracies of CTM and other commercialized PCR methods. Cobas Amplicor was one of the most commonly used commercialized PCR system until the Cobas TaqMan system became available^5^,^6^,^7^. According to three large-scale studies that evaluated the diagnostic accuracy of Amplicor, the sensitivity ranged from 93% to 98% for the smear-positive specimens and from 40% to 62% for the smear-negative specimens; and the specificity ranged from 75% to 91% for the smear-positive specimens and from 99% to 99% the smear-negative specimens. According to these existing reports and the current meta-analysis, CTM has the better specificity for the smear-positive specimens. Xpert MTB/RIF is another currently available PCR technology, whose high performance is supported by heminested real-time PCR technology and single-use cartridge system with low risk of contamination^13^. Although a number of studies concerning the diagnostic accuracy of Xpert MTB/RIF have been reported since 2010, very few head-to-head comparative studies have been conducted for CTM and Xpert MTB/RIF^13^,^14^,^15^. Despite inconsistency, these head-to-head comparisons suggested that CTM and Xpert MTB/RIF had the generally equivalent diagnostic accuracy^13^,^14^,^15^. Therefore, key features for Xpert MTB/RIF compared to CTM are ability to detect resistance to rifampicin, simple procedure, and high cost. Thus, we believe Xpert MTB/RIF has good indication in middle/high income regions such as EU, where drug-resistant TB is prevalent. Loop-mediated isothermal amplification assay is also a recently developed gene amplification method^27^. In contrast to the PCR technology in which the reaction is carried out with a series of alternating temperature steps, isothermal amplification can be carried out at a constant temperature. The pooled sensitivity and the pooled specificity based on a meta-analysis published in 2014 were 80% (95% CI 78–83%), and 96% (95–97%), respectively^27^, which seems inferior to those by CTM in the current analysis. However, in the high-quality study subgroup, the pooled sensitivity and the pooled specificity was 90.0% (95% CI, 86.0–93.0%) and 99.0% (95% CI, 98.0–100.0%), respectively. Given the low cost and simplicity, these diagnostic accuracies of loop-mediated isothermal amplification assay are acceptable, especially in the developing countries. The real-time PCR, developed two decades earlier, measures PCR product accumulation by a dual-labeled fluorogenic TaqMan Probe. Unlike the conventional quantitative PCR methods, the real-time PCR does not demand post-PCR sample handling. This prevents potential PCR product carry-over contamination and results in a faster and higher throughput analyses. Therefore, the real-time PCR can provide the accurate and reproducible results^28^.

The primary limitations of the current meta-analyses stemmed from the study design of each original study. The non-blinded examinations and the non-prospective study designs usually impair the quality of a study for a diagnostic test accuracy^18^. However, these factors probably may not have considerably affected our results, as both the reference culture and the index CTM usually provide the inflexible non-subjective results. Another limitation was the possibility of the false negatives from the reference test, the TB culture^2^,^3^. One strategy to account for the culture false negative is introducing a composite reference standard combining the results of several imperfect references^29^. Although we acknowledged the merit of the composite reference standard for TB diagnosis, we could not use this for the current meta-analysis because most researchers frequently used the non-composite culture as the simple golden standard. The third possible limitation is that we did not consider the interaction between CTM and HIV status, which potentially affected the results of CTM. Reviewing nature of the study also might be a possible limitation.

In conclusion, based on the meta-analysis using the bivariate model and the sufficient number of specimens, the diagnostic accuracy of CTM for the respiratory specimens was excellent. The summary estimates sensitivities and specificities using for the culture proven TB as reference test were 0.952 and 0.916 for the smear positive specimens and 0.600 and 0.989 for the smear negative specimens, respectively. The summary estimate sensitivity and specificity were 0.586 and 0.984 for the non-respiratory specimens, respectively. We believe that the information obtained from this study will aid the decision making of physicians who take care of patients with possible *M. tuberculosis* infection.

## Additional Information

**How to cite this article**: Horita, N. *et al.* Sensitivity and specificity of Cobas TaqMan MTB real-time polymerase chain reaction for culture-proven Mycobacterium tuberculosis: meta-analysis of 26999 specimens from 17 Studies. *Sci. Rep.* **5**, 18113; doi: 10.1038/srep18113 (2015).

## Supplementary Material

Supplementary Information

## Figures and Tables

**Table 1:** Characteristics of the included studies.

Author Year	Country (Income class)	Recruitment	Study design	Acid-fast stain	Specimen preparation for Cobas TaqMan	Culture	TB confirmation	Respiratory specimen	Non-respiratory specimen	Specimen number
Antonenka 2013	Germany (A)	Specimen bank	R, F, I	AR	NALC-NaOH	MGIT, LJ	Genotype CM, Genotype MTBC	sp, ba		R 116 NR 0
Bloemberg 2013	Switzerland (A)	Tertiary care diagnostic center	P, I	AR, ZN	NALC-NaOH	MGIT, 7H11	16SrRNA	sp, bal, ba, other	ti, ur, csf, ln, as, ab	R 829 NR 280
Causse 2011	Spain (A)	?		AR	NALC-NaOH	7H9, LJ, liquid medium	?		csf, pf, ar, as, ln, ti, gf	R 0 NR 340
Chandran 2010	India (C)	Tb Control Program	Short article	?	NALC-NaOH	LJ	Genotype MTBC	sp		R 72 NR 0
Cho 2015	Korea (A)	University hospital	I	?	NALC-NaOH	2%Ogawa, MGIT	multiplex PCR	sp, ba, bl, bal, ts	pus, pf, csf, ur, ti, ab	R 2384 NR 626
Choi 2013	Korea (A)	?	F, Korean, #1	AR, ZN	NALC-NaOH	MGIT, 3%Ogawa	Seeplex	sp, bal		R 619 NR 0
Huh 2015	Korea (A)	Tertiary care hospital	R #2	AR, ZN	NALC-NaOH (2%)	Liquid, solid medium	cordF, MPT64, MTB-ID V3	?		R 6772 NR 0
Ikegame 2012	Japan (A)	?	R	AR	NaOH	?	?	sp, bal, bl		R 1745 NR 0
Jönsson 2015	Sweden (A)	University hospital laboratory	R, I	?	NALC-NaOH	LJ, MGIT	AccuProbe, 16SrRNA GenoType MTBC,	sp, bal, bl	bf, ti, ur, gf, csf	R 2388 NR 1005
Kim JH 2011	Korea (A)	Tertiary care hospital	P, I, #2	ZN	NALC-NaOH	3%Ogawa	Amplicor, history	sp, bal, pf	ar, as, bf, ti	R 92 NR 0
Kim JW 2013	Korea (A)	?	F, #1	?	NALC-NaOH	MGIT	PCR assay Smear	bal, ba, sp	ab, csf, ar, as, pf, ti, ur	R 360 NR 65
Lee 2013	Taiwan (B)	University hospital & General hospital	P, I	AR, Ki	NALC-NaOH	MGIT, LJ	Middlebrook7H11	sp		R 586 NR 0
Lim 2014	Korea (A)	University hospital	P, B	ZN	NALC-NaOH (4%)	3%Ogawa	?	sp, bal		R 1167 NR 0
Linasmita 2012	Thailand (B)	University hospital	P, I	?	QIAGEN	MGIT	?		cervical ln	R 0 NR 73
Park 2013	Korea (A)	Terciary care hospital	P, B, #2	AR,ZN	NALC-NaOH (2%)	MGIT, 3%Ogawa	cord F, conventional PCR MPT64, MTB-ID V3,	sp, bal		R 320 NR 0
Tortoli 2012	Italy (A)	Reference center laboratory	R	AR	NALC-NaOH (1%)	MGIT, LJ	GenoType, 16SrRNA	bl, gf, sp	ti, bf, csf, pf, ab, ur	R 4340 NR 1727
Yang 2011	Taiwan (B)	University hospital laboratory	P	AR, Ki	NALC-NaOH	LJ, MGIT, 7H11	MPT64, GenoType, Mycobacterium CM assay	su, ba, bal		R 1093 NR 0

Income class: The World Bank income classification. A, high-income economy; B, upper-middle-income economy; C, lower-middle-income economy; D, low-income economy. Taiwan was classified as China for this table.

Study design: P, prospective study; R, retrospective study; F, using frozen specimen; B, TaqMan MTB was conducted blindly; I, invalid rate was reported; if these information was not provided, left blank. Short article and non-English article were also mentioned. #1, Choi and Kim JW used data from same hospital in different time. #2, Huh, Kim JH, and Park used data from same hospital in different time.

Acid-fast stain: ZN, Ziehl-Neelsen; AR, auramine-rhodamine; Ki, Kinyoun.

Specimen preparation for Cobas TaqMan: NALC-NaOH, N-acetyl-l-cysteine with sodium hydroxide; NaOH, sodium hydroxide.

Culture: MGIT, Mycobacteria Growth Indicator Tube; LJ, Löwenstein-Jensen; 7H9, Middlebrook 7H9 Broth; 7H10/11, Middlebrook 7H10/11 Agar; BacT/A, BacT/Alert.

Tb confirmation: 16SrRNA, 16S rRNA gene sequence; cordF, cord formation.

Respiratory specimen: sp, sputum; ba, broncheal/tracheal aspirate; bl, broncheal/tracheal lavage; bal, bronchialalveolar lavage; gf, gastric fluid; ts, throat swab.

Non-respiratory specimen: ln, lymph node; pf, pleural fluid; ar, articular fluid; as, ascite fluid; ab, abcess/pus; ur, urine; csf, cerebrospinal fluid; bf, body fluid; bl, blood; gf, gastric fluid; ti, other tissue sample.

Specimen number: R, number of the respiratory specimens; NR, number of the non-respiratory specimens.

**Table 2:** Summary of the results.

Specimen type	Study n	Specimen n	Sensitivity (95% CI)	Specificity (95% CI)	DOR (95% CI), I^2^	AUC
Respiratory	15	22883	0.808 (0.758–0.850)	0.990 (0.981–0.994)	459 (261–805), 26%	0.956
Respiratory, smear+	7	821	0.952 (0.926–0.969)	0.916 (0.797–0.968)	269(66–1104), 0%	0.961
Respiratory, smear–	7	11621	0.600 (0.459–0.726)	0.989 (0.981–0.993)	132 (71–243), 14%	0.971
Non–respiratory	7	4116	0.586 (0.437–0.721)	0.984 (0.955–0.994)	86 (34–217), 7%	0.898

DOR: diagnostic odds ratio. I^2^: Higgins’ heterogeneity. AUC: area under receiver operating characteristics curve.
