# Supplementary material for: Sensitivity and specificity of Cobas TaqMan MTB real-time polymerase chain reaction for culture-proven Mycobacterium tuberculosis: meta-analysis of 26999 specimens from 17 Studies
Source: Sci Rep. 2015 Dec 9;5:18113. doi: 10.1038/srep18113 (PMC4673449; doi:10.1038/srep18113)

**Sensitivity and specificity of Cobas TaqMan MTB real-time polymerase chain reaction**

**for culture-proven Mycobacterium tuberculosis:**

**meta-analysis of 26999 specimens from 17 Studies.**

**<<Supplementary Data>>**

**Authors:**

^1)^Nobuyuki Horita, ^1)^Masaki Yamamoto, ^1)^Takashi Sato, ^1)^Toshinori Tsukahara, ^1)^Hideyuki Nagakura, ^1)^Ken Tashiro, ^1)^Yuji Shibata, ^1)^Hiroki Watanabe, ^1)^Kenjiro Nagai, ^1)^Kentaro Nakashima, ^1)^Ryota Ushio, ^1)^Misako Ikeda, ^2)^Kentaro Sakamaki, ^3)^Takashi Yoshiyama, ^1)^Takeshi Kaneko

^1)^Department of Pulmonology, Yokohama City University Graduate School of Medicine. 3-9, Fukuura, Kanazawa, Yokohama, Japan.

^2)^Department of Biostatistics, Yokohama City University Graduate School of Medicine. 3-9, Fukuura, Kanazawa, Yokohama, Japan.

^3)^Division of Respiratory Medicine, Japan-Anti-Tuberculosis Association Fukujuji Hospital. 3-1-24, Matsuyama, Kiyose, Tokyo, Japan.

**Supplementary Text 1. List of included original studies.**

We included following 17 original studies for the current meta-analysis.

1 Antonenka U., Hofmann-Thiel S., Turaev L., Esenalieva A., Abdulloeva M., et al. Comparison of Xpert MTB/RIF with ProbeTec ET DTB and COBAS TaqMan MTB for direct detection of M. tuberculosis complex in respiratory specimens. *BMC Infect Dis.* **13**:280 (2013).

2 Bloemberg G. V, Voit A., Ritter C., Deggim V., & Böttger E. C. Evaluation of Cobas TaqMan MTB for direct detection of the Mycobacterium tuberculosis complex in comparison with Cobas Amplicor MTB. *J Clin Microbiol.* **51**:2112-2117 (2013).

3 Causse M., Ruiz P., Gutiérrez-Aroca J. B., & Casal M. Comparison of two molecular methods for rapid diagnosis of extrapulmonary tuberculosis. *J Clin Microbiol.* **49**:3065-3067 (2011).

4 Chandran S. P., & Kenneth J. Evaluation of COBAS TaqMan real time PCR assay for the diagnosis of Mycobacterium tuberculosis. *Indian J Med Res.* **132**:100-102 (2010).

5 Cho W. H., Won E. J., Choi H. J., Kee S. J., Shin J. H. et al. Comparison of AdvanSure TB/NTM PCR and COBAS TaqMan MTB PCR for Detection of Mycobacterium tuberculosis Complex in Routine Clinical Practice. *Ann Lab Med.* **35**:356-361 (2015).

6 Choi J. C. Performance of Cobas TaqMan MTB for detection of mycobacterium tuberculosis complex in respiratory specimens. *Soonchunhyang Med Sci.* **19**:1-5 (2013). [Korean]

7 Huh H. J., Koh W.J., Song D.J., Ki C.S., & Lee N. Y. Evaluation of the Cobas TaqMan MTB test for the detection of Mycobacterium tuberculosis complex according to acid-fast-bacillus smear grades in respiratory specimens. *J Clin Microbiol.* **53**:696-698 (2015).

8 Ikegame S., Sakoda Y., Fujino N., Taguchi K., Kawasaki M., & et al. Clinical Evaluation of COBAS TaqMan PCR for the Detection of Mycobacterium tuberculosis and M. avium Complex. *Tuberc Res Treat.* **2012**:170459 (2012).

9 Jönsson B., Lönnermark E., & Ridell M.. Evaluation of the Cobas TaqMan MTB test for detection of Mycobacterium tuberculosis complex. *Infect Dis.* **47**:231-236 (2015).

10 Kim J. H., Kim Y. J., Ki C. S., Kim J. Y., & Lee N.Y. Evaluation of Cobas TaqMan MTB PCR for detection of Mycobacterium tuberculosis. *J Clin Microbiol.* **49**:173-176 (2011).

11 Kim J. W., Choi Y. J., Kim H. J., Park J. S., Nam H. S., et al. Comparison of PNA probe-based real-time PCR and Cobas TaqMan MTB for detection of MTBC. *BioChip J.* **7**:85-88 (2013).

12 Lee M. R., Chung K. P., Wang H. C., Lin C. B., Yu C. J., et al. Evaluation of the Cobas TaqMan MTB real-time PCR assay for direct detection of Mycobacterium tuberculosis in respiratory specimens. *J Med Microbiol* **62:**1160-1164 (2013).

13 Lim J., Kim J., Kim J. W., Ihm C., Sohn Y. H., et al. Multicenter evaluation of Seegene Anyplex TB PCR for the detection of Mycobacterium tuberculosis in respiratory specimens. *J Microbiol Biotechnol.* **24**:1004-1007 (2014).

14 Linasmita P., Srisangkaew S., Wongsuk T., Bhongmakapat T., & Watcharananan S. P. Evaluation of real-time polymerase chain reaction for detection of the 16S ribosomal RNA gene of Mycobacterium tuberculosis and the diagnosis of cervical tuberculous lymphadenitis in a country with a high tuberculosis incidence. *Clin Infect Dis.* **55**:313-321 (2012).

15 Park K. S., Kim J.Y., Lee J.W., Hwang Y. Y., Jeon K., et al. Comparison of the Xpert MTB/RIF and Cobas TaqMan MTB assays for detection of Mycobacterium tuberculosis in respiratory specimens. *J Clin Microbiol.* **51**:3225-3227 (2013).

16 Tortoli E., Urbano P., Marcelli F., Simonetti T.M., & Cirillo D.M. Is real-time PCR better than conventional PCR for Mycobacterium tuberculosis complex detection in clinical samples? *J Clin Microbiol.* **50**:2810-2813 (2012).

17 Yang Y. C., Lu P. L., Huang S. C., Jenh Y.S., & Jou R. Evaluation of the Cobas TaqMan MTB test for direct detection of Mycobacterium tuberculosis complex in respiratory specimens. *J Clin Microbiol.* **49**:797-801 (2011).

**Supplementary Table 1. Number of the specimens evaluated for diagnostic accuracy.**

|  | Respiratory specimens | | | |  | | | |  | | | | Non-respiratory specimens | | | |
| --- | --- | --- | --- | --- | --- | --- | --- | --- | --- | --- | --- | --- | --- | --- | --- | --- |
|  | Any smear type | | | | Smear positive subgroup | | | | Smear negative subgroup | | | | Any smear type | | | |
|  | TP | FP | FN | TN | TP | FP | FN | TN | TP | FP | FN | TN | TP | FP | FN | TN |
| Antonenka 2013 | 48 | 0 | 17 | 51 | 14 | 0 | 1 | 23 | 34 | 0 | 16 | 28 |  |  |  |  |
| Bloemberg 2013 | 61 | 9 | 8 | 751 |  |  |  |  |  |  |  |  | 14 | 14 | 8 | 244 |
| Causse 2011 |  |  |  |  |  |  |  |  |  |  |  |  | 32 | 5 | 9 | 294 |
| Chandran 2010 | 35 | 1 | 0 | 36 |  |  |  |  |  |  |  |  |  |  |  |  |
| Cho 2015 | 122 | 35 | 50 | 2,177 |  |  |  |  |  |  |  |  | 6 | 8 | 12 | 600 |
| Choi 2013 | 38 | 0 | 9 | 572 |  |  |  |  |  |  |  |  |  |  |  |  |
| Huh 2015 | 180 | 32 | 89 | 6,471 | 108 | 5 | 2 | 127 | 72 | 27 | 87 | 6,344 |  |  |  |  |
| Ikegame 2012 | 153 | 6 | 24 | 1,562 |  |  |  |  |  |  |  |  |  |  |  |  |
| Jönsson 2015 | 173 | 12 | 80 | 2,123 | 90 | 0 | 0 | 25 | 83 | 12 | 80 | 2,098 | 65 | 9 | 63 | 868 |
| Kim JH 2011 | 19 | 3 | 5 | 65 |  |  |  |  |  |  |  |  |  |  |  |  |
| Kim JW 2013 | 97 | 0 | 20 | 243 |  |  |  |  |  |  |  |  | 3 | 0 | 10 | 52 |
| Lee 2013 | 182 | 13 | 38 | 353 | 167 | 7 | 10 | 25 | 15 | 6 | 28 | 328 |  |  |  |  |
| Lim 2014 | 81 | 21 | 7 | 1,058 | 59 | 3 | 3 | 8 | 22 | 18 | 4 | 1,050 |  |  |  |  |
| Linasmita 2012 |  |  |  |  |  |  |  |  |  |  |  |  | 23 | 4 | 6 | 40 |
| Park 2013 | 20 | 6 | 8 | 286 | 13 | 0 | 2 | 11 | 7 | 6 | 6 | 275 |  |  |  |  |
| Tortoli 2012 | 158 | 2 | 37 | 4,143 |  |  |  |  |  |  |  |  | 36 | 2 | 20 | 1,669 |
| Yang 2011 | 129 | 12 | 12 | 940 | 94 | 0 | 3 | 21 | 35 | 12 | 9 | 919 |  |  |  |  |

TP: true positive. FP: false positive. FN: false negative. TN: true negative.

**Supplementary Figure 1. Quality Assessment of Diagnostic Accuracy Studies-2 (QUADAS2) assessment: risk of bias summary.**


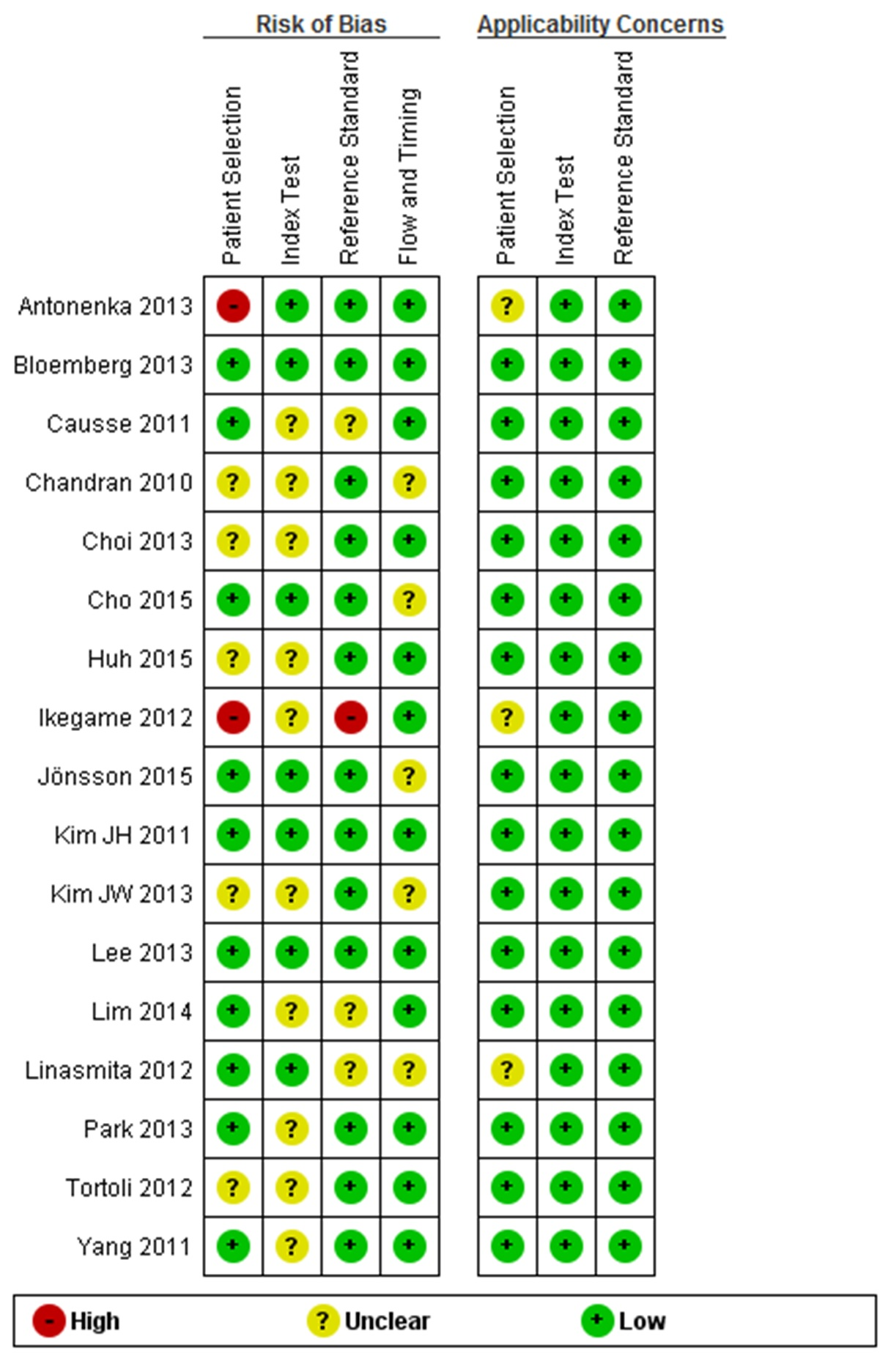

Supplement: Supplementary Information [file srep18113-s1.doc]
